# Influence of Preclinical Medical Scribe Experiences on Specialty Outcomes in Fourth-year Medical Students

**DOI:** 10.1007/s40670-025-02571-w

**Published:** 2025-11-18

**Authors:** Kevin Chao, Kathryn Veazey

**Affiliations:** 1 Drexel University College of Medicine, Philadelphia, PA USA; 2 Neurobiology and Anatomical Sciences, Drexel University College of Medicine, Wyomissing, PA USA

**Keywords:** Medical scribe experience, Specialty selection process, Residency match, Professional development, Career exploration

## Abstract

Many medical students pursue pre-matriculation medical scribe experiences. Previous research has explored how these experiences may benefit academic performance, confidence, and commitment to medicine. However, no studies have investigated the effect of medical scribe experiences on specialty selection outcomes. Seven fourth-year medical students were interviewed after matching through the National Residency Matching Program in 2023 and 2024. Interviews revealed that scribing had minimal impact on student specialty selection, but significantly influenced career exploration, personal growth, and professional development.

## Background

Medical scribes are becoming increasingly prevalent in the age of the electronic health record and serve multiple roles in the healthcare industry [1]. Previous research has investigated the impact of medical scribes on physician workflow via patient and physician satisfaction, productivity, and documentation burden, but there is less information available about the personal impact of medical scribing [2–8].

Medical scribe experiences may support matriculation into medical school [9, 10]. Once matriculated, the effects of scribe experiences on Step 1 and Step 2 scores, preclinical performance, commitment to medicine, and degree of confidence in clinical skills have been well-documented [11–14]. Yet there exists a gap in knowledge regarding the impact of medical scribe experience on specialty selection outcomes.

This exploratory study aimed to describe the effect of medical scribe experiences on senior medical students regarding specialty selection, clinical preparation, and readiness for medical school.

### Activity

The study utilized an interpretive phenomenological approach using semi-structured interviews. The script was informed by the tenets of social cognitive career theory [15]. Throughout the research study, the student interviewer remained aware and reflective of how personal biases might influence the interview and analysis process.

Students were recruited from the Drexel University College of Medicine (DUCOM) from the classes of 2023 and 2024. Students were eligible if they had at least six months of consecutive experience as a medical scribe, during which they worked at least 35 h per week before matriculating.

Interviews were conducted virtually via Zoom by KC. This study was approved by DUCOM’s Institutional Review Board (IRB #2206009313). Informed consent was obtained before interviews. All published information has been de-identified.

The transcripts underwent thematic analysis [16, 17]. An initial codebook was generated after familiarization with a subset of transcripts and modified with each additional transcript. The final codebook was applied to the initial transcripts, then used to construct themes. Coding was managed using Dedoose v10.0.25 [18].

## Results & Discussion

### Description of the Sample

An initial recruitment survey yielded 23 responses, of which seven participated in interviews (4 - class of 2023; 3 - class of 2024). Interviews lasted between 19 min and 36 min. One participant verified their transcript.

Most participants identified as female (*n* = 4), White (*n* = 4), and non-Hispanic (*n* = 7). Participants scribed across nine specialties. Two participants matched into their scribed specialty, and all participants matched into their preferred specialty of choice.

### Results of Thematic Analysis

#### Theme 1: Becoming Acquainted with Medicine

Participants described formative experiences that provided early, low-risk exposure to medicine. Scribing allowed them to passively observe healthcare delivery and better understand physicians’ roles while considering their career choices. Scribing also exposed participants to many diverse patient populations, which helped shape their professional goals and encouraged some to pursue careers in medicine.

#### Theme 2: Accumulating Social Capital

Through interactions with physicians and patients, participants strengthened their interpersonal skills and better envisioned themselves in medical roles. They established professional relationships that offered mentorship, leadership, and academic opportunities, which many thought enhanced their medical school and residency applications.

#### Theme 3: Committing to Medicine

Scribing helped participants assess how their interests, values, and abilities aligned with medical practice. These experiences encouraged introspection, self-reflection, and personal growth, which heightened their commitment to medicine, regardless of matched specialty.

#### Theme 4: Committing to a Specialty

**Fig. 1:**
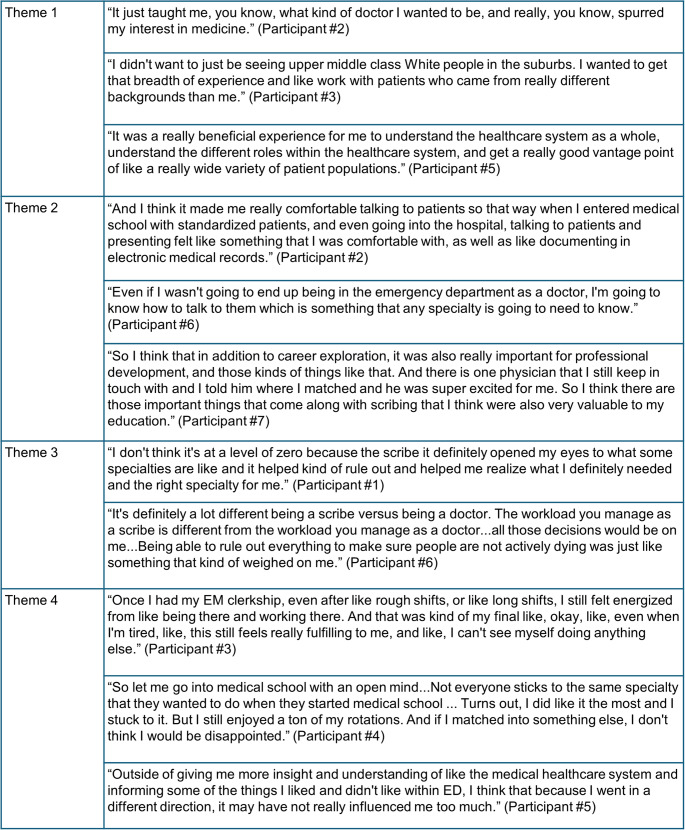
Supportive Quotes for Themes

While scribing offered many benefits, participants transitioned from observers to active practitioners during clerkships. These experiences reinforced insights established while scribing and solidified many participants’ specialty choices. Despite this, some participants felt that their scribing experiences had little to no impact on their specialty selection journey (Fig 1).

## Discussion

Prior scribe experiences had limited direct impact on specialty choices among fourth-year medical students, but indirectly enabled participants to expand their professional networks, develop clinical skills, explore different specialties, and prepare for careers in medicine. These early experiences were reinforced in the clerkships, which more strongly influenced specialty decisions.

Participants noted that scribing reframed their perceptions of certain specialties by revealing their day-to-day realities. These insights into various lifestyle factors may have indirectly influenced participants’ specialty decisions, as seen in previous studies [19, 20]. While most did not match into their scribed specialty, scribing supported early career exploration, which may provide a potential avenue to address projected healthcare shortages by increasing interest in certain medical pathways [21]. Scribing also enhanced social capital - defined as the ways that communities build relationships, establish shared norms, and engage in shared activities [22]. Lastly, it fortified many participants’ commitment to medicine, despite the extraordinary challenges faced by the healthcare field [13].

A significant limitation of this study is the small sample size, which may affect the generalizability, but can serve as an initial pilot study for future, larger research projects. Other limitations include the absence of pilot interviews, field notes, member checks, and data saturation. As data were collected from a single institution, findings may not be transferable. Further research is encouraged, especially on the experiences of scribes from underrepresented, first-generation, rural, or low-income backgrounds. Future work might apply grounded theory informed by various foundational theories, like situated learning and legitimate peripheral participation [23].

## Data Availability

The datasets generated during and/or analyzed during the current study are available from the corresponding author on reasonable request.
